# Cardiovascular Aging and Damage in Patients with Iron Overload

**DOI:** 10.3390/biomedicines14071487

**Published:** 2026-06-30

**Authors:** Marcin Gruszecki, Krzysztof Młodziński, Michał Świątczak, Agnieszka Gruszecka, Adam Bujnowski, Anna Lewandowska, Stanisław Karmoliński, Damian Kaufmann, Ludmiła Daniłowicz-Szymanowicz

**Affiliations:** 1 Department of Radiology Informatics and Statistics, Faculty of Health Sciences, Medical University of Gdansk, Tuwima 15, 80-210 Gdansk, Poland; 2 Department of Biomedical Engineering, Faculty of Electronics, Telecommunications and Informatics, Gdansk University of Technology, Narutowicza 11/12, 80-233 Gdansk, Poland; 3 Department of Cardiology and Electrotherapy, Faculty of Medicine, Medical University of Gdansk, Smoluchowskiego 17, 80-211 Gdansk, Poland

**Keywords:** iron overload, hereditary hemochromatosis, cardiovascular aging, cardiovascular variability, wavelet transformation, phase coherence, autonomic dysfunction, blood pressure variability, electrocardiography, oxidative stress

## Abstract

**Introduction**: Vascular aging is characterized by endothelial dysfunction, impaired vasomotor regulation, and structural remodeling. Iron overload may accelerate these processes through oxidative stress, but its effects on cardiovascular regulation remain incompletely understood. **Methods**: We investigated cardiovascular dynamics in patients with hereditary hemochromatosis using wavelet-based time–frequency analysis of electrocardiographic and blood pressure signals. Continuous beat-to-beat recordings were analyzed to assess oscillatory patterns and phase coherence across physiologically relevant frequency bands. **Results**: Patients with iron overload exhibited significant alterations compared with healthy controls, including reduced cardiac-related variability, increased peripheral blood pressure oscillations, and disrupted phase coherence between cardiac and vascular signals. These findings indicate impaired integration between central cardiac activity and peripheral vascular regulation. **Conclusions**: Iron overload is associated with early cardiovascular dysregulation, likely driven by autonomic imbalance and vascular dysfunction. Wavelet-based metrics may enable sensitive detection of subclinical alterations and improve early risk stratification in patients with iron metabolism disorders.

## 1. Introduction

Vascular aging is a multifaceted physiological process that affects the entire circulatory system, from large arteries to the microcirculation. It is characterized by impaired vasomotor function, altered endothelial secretory activity, disrupted intercellular communication, structural vascular remodeling, and compromised barrier function between the bloodstream and vascular smooth muscle layer [1]. Among the factors contributing to accelerated vascular aging, disturbances of iron metabolism, particularly systemic iron overload, have attracted increasing attention because of their capacity to promote oxidative stress and vascular injury [2,3]. However, the precise effects of iron overload on cardiovascular regulation remain incompletely understood, and available data are often limited or inconsistent. At the same time, growing evidence indicates that iron overload may contribute to both vascular dysfunction and cardiac abnormalities, suggesting that these processes may represent interconnected manifestations of cardiovascular injury [4,5,6,7].

In hereditary hemochromatosis (HH), excess iron accumulation has traditionally been considered the primary mechanism responsible for cardiac damage. Recent evidence suggests that iron-mediated toxicity may also involve indirect mechanisms related to oxidative stress, inflammation, and endothelial dysfunction [4,5,8]. In particular, non-transferrin-bound iron (NTBI) may promote reactive oxygen species (ROS) generation and cellular injury [9]. These processes have been linked to endothelial dysfunction, myocardial fibrosis, ventricular stiffening, and impaired cardiac function, potentially contributing to the development of heart failure and other cardiovascular complications [10,11,12].

Therefore, the aim of the present study was to investigate cardiovascular dynamics in hereditary hemochromatosis using wavelet-based time-frequency analysis of ECG and blood pressure signals. By comparing HH patients with both young and older healthy controls, we sought to distinguish alterations potentially associated with iron overload from those attributable to physiological aging and hypothesized that systemic iron overload would be associated with disturbances in cardiovascular oscillatory activity and cardiac–vascular coupling.

## 2. Materials and Methods

### 2.1. Subjects

Patient group: Thirty patients with genetically confirmed HFE-related hereditary hemochromatosis (HH) were enrolled in the study. Inclusion criteria comprised age ≥ 18 years and provision of written informed consent. Exclusion criteria included any previously diagnosed cardiovascular disease (except treated arterial hypertension), clinical evidence of cardiac involvement, or left ventricular ejection fraction (LVEF) < 50%. The HH cohort consisted of 20 men and 10 women with a mean age of 47 ± 13 years and a body mass index (BMI) of [29.1 ± 6.7] kg/m^2^. All participants were non-smokers. Sixteen patients (53%) were not receiving regular pharmacological treatment. Antihypertensive therapy, including angiotensin-converting enzyme inhibitors, was reported in 12 patients (40%), and statin therapy was reported in 14 patients (46%). Because these treatments may influence autonomic tone, vascular function, and blood pressure variability, medication use was considered an important limitation of the present exploratory analysis. Iron metabolism parameters, including serum ferritin and transferrin saturation, were collected as part of the clinical characterization of the HH cohort and are summarized in Table 1.

Control groups: Two healthy control groups were included: a young control group (16 males and 14 females; mean age 23 ± 1 years; BMI 22.9 ± 2.1 kg/m^2^) and an older control group (18 males and 12 females; mean age 62 ± 8 years; BMI 26.4 ± 4.4 kg/m^2^). All control participants were non-smokers and free of known cardiovascular disease. The two control groups were included to distinguish alterations potentially associated with hereditary hemochromatosis from those attributable to physiological aging. Therefore, age matching between groups was not performed by design.

The study was conducted in accordance with the Declaration of Helsinki and was approved by the Ethics Committee of the Medical University of Gdansk (NBBN/452/2016). Written informed consent was obtained from all participants prior to enrollment.

### 2.2. Experimental Design

All patients and controls underwent continuous, non-invasive, beat-to-beat recording of systolic arterial pressure (SAP) and heart period (HP) using the Finapres NOVA system (Finapres Medical Systems, San Diego, CA, USA), with a cuff placed on the middle phalanx of the third finger of the right hand. The ECG ground electrode was positioned on the left anterior superior iliac spine, while the two primary leads were placed beneath the midpoints of the left and right clavicles [13]. Measurements were conducted in the morning in a quiet room, with participants from both the control and patient groups in a supine position. Each participant lay on a bed with a headrest for a 15 min stabilization period and was provided with a blanket if necessary. The room temperature was maintained at 18–20 °C, with minimal ambient lighting. Participants were instructed to abstain from food for at least 4 h and from smoking and caffeine for at least 12 h prior to the measurements. Strenuous physical activity was not permitted within 6 h of testing, and all participants were required to empty their bladder within 30 min before the session [14]. During the first 20 min after positioning, SAP and HP were monitored to confirm physiological stabilization. The device’s self-adjustment function was disabled immediately before data acquisition and reactivated for recalibration following each recording session.

#### 2.2.1. Measurements

The SAP and HP signals were recorded at 300 Hz and subsequently processed for analysis. Pre-processing included detrending and normalization, achieved by subtracting the mean and dividing by the signal’s standard deviation [15]. To reduce data volume while preserving key physiological features of the ECG and BP waveforms, the signals were downsampled to 20 Hz.

#### 2.2.2. Wavelet Transform

Wavelet analysis was used to identify and explore the physiological mechanisms driving oscillations within the cardiovascular system. As a robust signal processing technique, the wavelet transform converts signals from the time domain into the time-frequency domain. Wavelet amplitude reflects the strength (power) of oscillatory activity within a given frequency band, with higher values indicating a greater contribution of that oscillatory component to the analyzed signal. The wavelet transform is defined as follows:

$$W\left(s,t\right)=\frac{1}{\sqrt{s}}\int_{-\infty }^{+\infty }\varphi \left(\frac{u-t}{s}\right)g\left(u\right)du,$$

where *W*(*s*, *t*) is the wavelet coefficient, *g*(*u*) is the time series, and *φ* is the Morlet mother wavelet, scaled by factor *s* and translated in time by *t*. The Morlet mother wavelet is defined by the following equation:

$$\varphi (u)=\frac{1}{\sqrt[{4}]{\pi }}exp(-i2\pi u)exp(-0.5u^{2}),$$

where $i=\sqrt{-1}$.

The complex Morlet wavelet was selected because of its favorable time-frequency localization properties and its widespread use in the analysis of non-stationary physiological signals [16].

When applied in wavelet analysis, the Morlet wavelet produces complex-valued coefficients in the time-frequency domain:

$$X\left(\omega_{k},t_{n}\right)=X_{k,n}=a_{k,n}+ib_{k,n}.$$

They define the instantaneous relative phase,

$$\theta_{k,n}=arctan\left(\frac{b_{k,n}}{a_{k,n}}\right),$$

and the absolute amplitude,

$$\left|X_{k,n}\right|=\sqrt{a_{k,n}^{2}+b_{k,n}^{2}},$$

for each frequency and time.

During measurements, the heart may produce phase modulations. To assess the relationship between the phases of two signals, wavelet phase coherence (WPCO) is used. WPCO allows us to evaluate whether oscillations in the signals remain significantly correlated over time, making it a valuable tool for analyzing the complex dynamics of cardiac activity and its interaction with other physiological signals. The WPCO was estimated using the following formula [17]:

$$C_{\theta }\left(f_{k}\right)=\frac{1}{n}\left|\sum_{t=1}^{n}exp\left[i\left(\theta_{2k,n}-\theta_{1k,n}\right)\right]\right|,$$

where $\theta_{k,n}=arctan\left(\frac{b_{k,n}}{a_{k,n}}\right)$ is an instantaneous measure of phase at each time and frequency for both signals. WPCO approaches zero when two oscillations are unrelated, and their phase difference continuously changes over time. When two oscillations are related, their phase difference remains constant over time, and the wavelet phase coherence (WPCO) approaches 1.

### 2.3. Statistical Analysis

Continuous variables are presented as mean ± standard deviation (SD). Data normality was assessed using the Shapiro–Wilk test. Volunteers were categorized into three groups: young (Y), aged (A), and hereditary hemochromatosis (HH). Differences among groups were evaluated using one-way analysis of variance (ANOVA). When a significant overall group effect was observed, pairwise comparisons were performed using Tukey’s post hoc test. Statistical significance was defined as *p* < 0.05. Coherence values exceeding the 95th percentile of the surrogate distribution were considered statistically significant.

To assess the statistical significance of wavelet phase coherence, surrogate data testing was applied [18]. Intersubject surrogates were generated by pairing ECG and BP signals from different participants, thereby preserving general spectral characteristics while eliminating genuine within-subject physiological coupling [19]. The resulting surrogate distribution represented the null hypothesis of no true ECG-BP interaction. At each frequency, observed coherence values were compared with the surrogate distribution, and values exceeding its 95th percentile were considered statistically significant. This procedure reduces the risk of falsely identifying coherence caused by finite signal length or shared spectral properties rather than genuine physiological coupling.

## 3. Results

**Figure 1:**
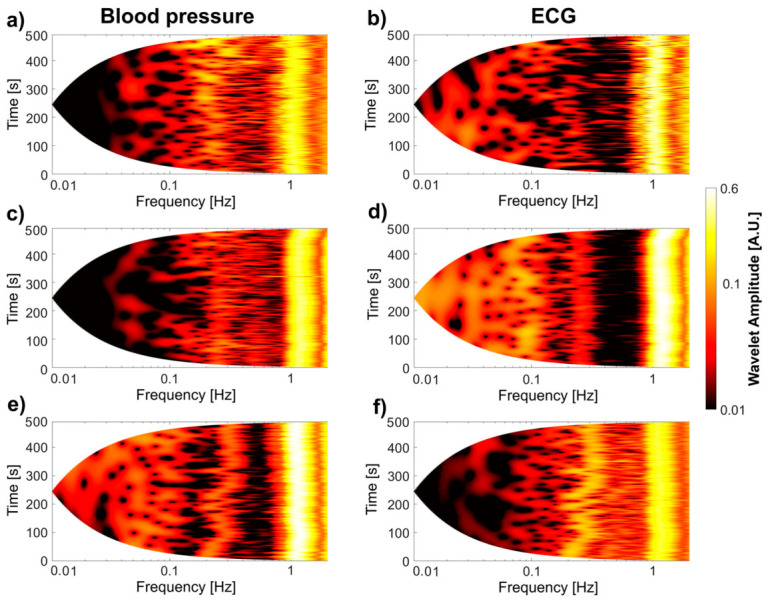
Wavelet amplitude of recorded signals: BP (**a**,**c**,**e**) and ECG (**b**,**d**,**f**) are shown for one representative volunteer. The top two panels (**a**,**b**) illustrate the results for one subject from the young healthy volunteer group, the middle two panels (**c**,**d**) display the results for one subject from the older people healthy volunteer group, and the bottom two panels (**e**,**f**) show the results for one subject from the hemochromatosis group.

Figure 1 presents representative wavelet transform (WT) amplitude maps obtained from BP and ECG recordings in a young healthy volunteer (panels a,b), an older healthy volunteer (panels c,d), and a patient with hereditary hemochromatosis (panels e,f). In all recordings, a prominent oscillatory component corresponding to cardiac activity is visible at approximately 1 Hz. Additional lower-frequency oscillations are also present, reflecting slower cardiovascular regulatory processes. Qualitatively, the HH subject exhibits a different distribution of oscillatory power across frequency bands compared with both control groups. These observations were subsequently evaluated quantitatively using group-level analyses.

**Figure 2:**
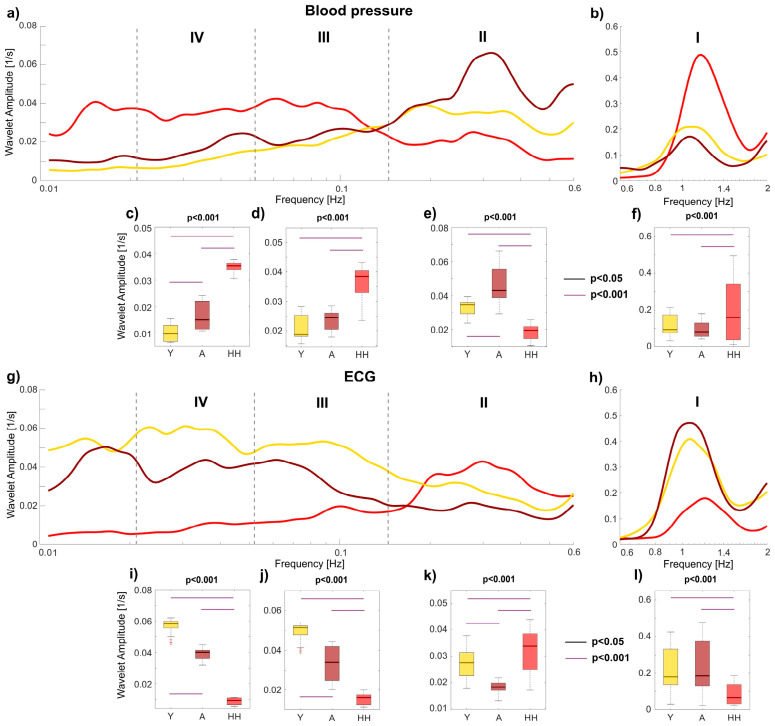
The median time-averaged wavelet transforms of BP (**a**,**b**) and ECG (**g**,**h**) signals were recorded from subjects in the hemochromatosis patient group and in both control groups. Box plots show the neurogenic (**c**,**i**), myogenic (**d**,**j**), respiratory (**e**,**k**), and cardiac (**f**,**l**) oscillations, as well as the wavelet amplitude in the BP signal (**c**,**d**,**e**,**f**) and ECG (**i**,**j**,**k**,**l**) within these four frequency intervals. The young (Y) group is represented in gold, the older people (A) group in brown, and the hemochromatosis (HH) patients in red.

Figure 2 summarizes the frequency-specific characteristics of BP and ECG signals based on median time-averaged WT amplitudes. Four physiologically relevant frequency intervals were analyzed according to the classification proposed by Stefanovska et al. [20] and Gruszecki et al. [15]: Intervals I (0.6–2 Hz) and II (0.145–0.6 Hz) are associated with cardiac and respiratory functions, respectively. Interval III (0.052–0.145 Hz) is linked to smooth muscle activity, while interval IV (0.021–0.052 Hz) is thought to reflect autonomic regulation of smooth muscle.

Compared with both control groups, patients with hereditary hemochromatosis demonstrated significant differences in oscillatory activity across the analyzed frequency bands. In general, BP-derived oscillations were increased in the HH group, whereas ECG-derived oscillations were reduced, indicating a dissociation between central cardiac activity and peripheral vascular dynamics. One-way ANOVA followed by Tukey’s post hoc testing confirmed significant differences between the HH group and both control groups in all analyzed frequency intervals (Figure 2c–f,i–l).

To further investigate the interaction between cardiac and vascular regulation, wavelet phase coherence was calculated between simultaneously recorded BP and ECG signals. A coherence value was considered statistically significant when it exceeded the 95th percentile of the surrogate distribution generated from 435 inter-subject surrogate pairs.

**Figure 3:**
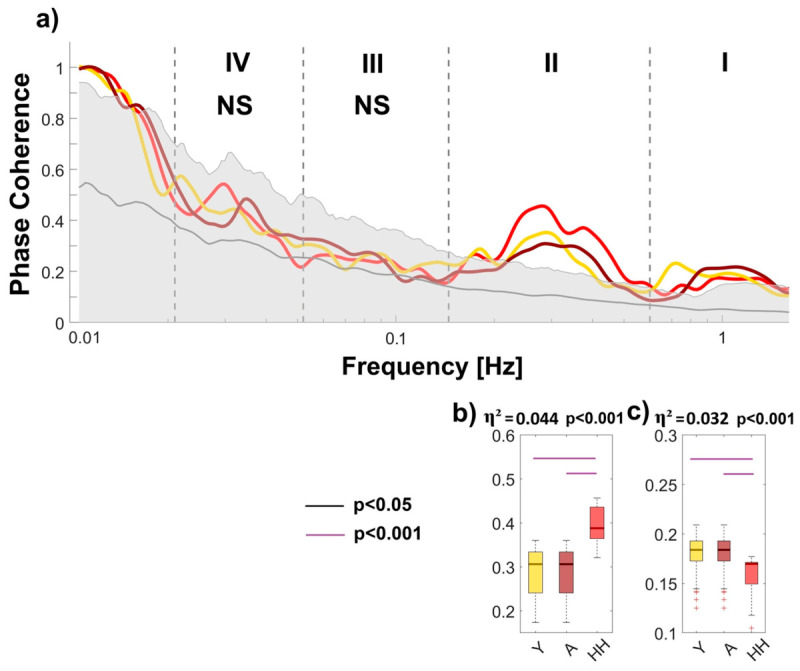
The median time-averaged phase coherence (**a**) estimated between BP and ECG signals are presented. Coherence below the 95th percentile of the surrogates is not considered significant and is shown by the light grey line and shading (**a**). Box plots display the respiratory (**b**), and cardiac (**c**) oscillations. The young (Y) group is represented in gold, the older people (A) group in brown, and the hemochromatosis (HH) patients in red. NS means not significant.

The median phase coherence spectrum is presented in Figure 3a together with the surrogate-derived significance threshold. Compared with both control groups, patients with hereditary hemochromatosis exhibited significantly lower coherence within the cardiac frequency band and significantly higher coherence within the respiratory frequency band (Figure 3b,c). No consistent group differences were observed in the lower-frequency neurogenic and myogenic ranges. These findings suggest altered cardiac–vascular coupling in hereditary hemochromatosis, particularly within frequency bands associated with cardiac and respiratory regulation.

To improve interpretation of the statistical findings, η^2^ effect sizes were reported for all one-way ANOVA analyses in Figure 2 and Figure 3. All ANOVA tests showed significant group effects (*p* < 0.001). For BP wavelet amplitudes (Figure 2c–f), η^2^ ranged from 0.019 to 0.168, indicating small to large effects depending on the frequency band. For ECG wavelet amplitudes (Figure 2i–l), η^2^ ranged from 0.125 to 0.352, indicating mainly medium to large group-related differences. For ECG-BP phase coherence (Figure 3b,c), η^2^ ranged from 0.032 to 0.044, indicating smaller but consistent group-related differences. Tukey post hoc comparisons with 95% confidence intervals supported the pairwise group differences, as the intervals did not cross zero.

Overall, patients with hereditary hemochromatosis exhibited lower ECG-derived oscillatory amplitudes, higher BP-derived oscillatory amplitudes, and altered ECG-BP phase coherence compared with both control groups. These differences were observed consistently across multiple frequency bands and support the presence of altered cardiovascular signal dynamics in the HH cohort.

## 4. Discussion

Our findings suggest disturbed cardiac–vascular interactions in hereditary hemochromatosis, reflected by reduced ECG oscillatory activity and enhanced peripheral BP variability. Reduced ECG activity may be consistent with previously reported subclinical myocardial abnormalities in HH [4], whereas amplified BP oscillations suggest impaired vascular regulation. Iron-mediated endothelial dysfunction, altered vasomotor responsiveness, and reduced baroreflex buffering may contribute to the increased peripheral BP variability observed in the HH group [21].

Respiratory-related oscillatory patterns provided additional evidence of altered cardiovascular regulation. Reduced oscillatory activity within the respiratory frequency band may reflect impaired tissue perfusion and compensatory respiratory activation. However, because respiratory function, gas exchange, and tissue oxygenation were not measured directly, these interpretations remain hypothesis-generating. Previous studies have shown that advanced iron-overload cardiomyopathy may impair gas exchange and trigger compensatory respiratory responses, which could contribute to the respiratory-related oscillatory changes observed in the present study [22].

The observed alterations are broadly consistent with established mechanisms of iron cardiotoxicity. Iron-mediated oxidative stress promotes myocardial injury, endothelial dysfunction, and structural remodeling, which may impair both cardiac performance and vascular regulation [10,23,24,25,26,27,28]. Consistent with this concept, we observed enhanced vasomotor oscillatory activity in the BP signal, suggesting increased vascular smooth muscle activation and vasoconstrictor drive in patients with hemochromatosis.

We also observed a divergence between ECG- and BP-derived variability within the 0.021–0.052 Hz frequency band, characterized by reduced ECG oscillatory power but enhanced BP oscillations. This pattern may reflect impaired cardiac autonomic responsiveness together with augmented peripheral sympathetic and vasomotor modulation [12,29,30,31,32]. Furthermore, patients with hereditary hemochromatosis demonstrated reduced ECG-BP phase coherence in the cardiac band and increased coherence in the respiratory band compared with both control groups. At the cardiac frequency, ECG-BP phase coherence reflects the temporal stability with which cardiac electrical activation is translated into the peripheral arterial pressure pulse, a process influenced by electromechanical coupling, pre-ejection period variability, pulse transit time, arterial stiffness, and vascular tone [33,34]. Reduced coherence may therefore indicate less stable electromechanical coupling and pulse transmission in the setting of iron-related myocardial and vascular dysfunction [35,36,37]. In contrast, increased coherence within the respiratory band may reflect stronger respiratory modulation of cardiovascular dynamics, including respiratory sinus arrhythmia and breathing-related fluctuations in venous return, stroke volume, and arterial pressure [38,39]. However, because respiratory parameters were not recorded directly, this interpretation should be regarded as hypothesis-generating.

Taken together, the present findings support the presence of altered cardiovascular signal dynamics in the studied HH cohort. However, because of the cross-sectional design, medication use, comorbidities, and the lack of direct myocardial iron, respiratory, and oxygen-transport measurements, the data cannot establish that systemic iron overload caused the observed alterations. Wavelet-derived amplitude and coherence measures should therefore be interpreted as exploratory tools for phenotyping cardiovascular regulation and generating hypotheses for future longitudinal, controlled studies.

Alternative conceptual frameworks linking cerebral energy metabolism and cardiovascular regulation may provide additional perspectives for interpreting cardiovascular dysregulation in iron-overload disorders, although these mechanisms were not investigated in the present study [38,39,40].

### Clinical Implications

If confirmed in larger and medication-sensitive cohorts, the observed dissociation between ECG-derived activity and BP-derived oscillations may support multimodal cardiovascular assessment in patients with HH, including electrophysiological evaluation and beat-to-beat blood pressure monitoring. Future studies should combine these recordings with direct myocardial iron quantification, for example, T2* cardiac MRI, baroreflex/autonomic assessment, and respiratory measurements. The present data do not justify specific therapeutic recommendations beyond standard management of hereditary hemochromatosis and cardiovascular risk factors. The elevated ferritin and transferrin saturation values observed in the HH cohort confirm the presence of systemic iron overload and support the biological plausibility of the cardiovascular alterations observed in this study. However, these parameters cannot directly quantify myocardial iron accumulation, and direct assessment of myocardial iron deposition was not available.

## 5. Conclusions

In this exploratory cross-sectional study, patients with hereditary hemochromatosis showed altered cardiovascular oscillatory activity and ECG-BP phase coherence compared with young and older controls. These findings suggest changes in cardiovascular regulation in the studied HH cohort, but they do not establish causality, medication-independent effects, direct links to myocardial iron burden, or conclusions regarding oxygen delivery. Wavelet-based analysis may help characterize subclinical cardiovascular signal abnormalities in future studies incorporating larger cohorts, direct iron quantification, respiratory measurements, and medication-sensitive designs.

## 6. Limitations

Several limitations of the present study should be acknowledged. First, the study was based on a relatively small sample size (30 patients with hereditary hemochromatosis and 30 participants in each control group), and the groups were not fully matched with respect to age and BMI. Both factors may independently influence autonomic regulation, vascular function, and blood pressure variability. Consequently, some of the observed differences in wavelet-derived amplitudes and ECG-BP phase coherence may reflect the combined and potentially interacting effects of iron overload, aging, and body composition.

Second, the present conclusions are based on alterations in cardiovascular signal characteristics and therefore remain indirect. Although the observed patterns are physiologically plausible and consistent with current knowledge of iron-mediated cardiovascular injury, causal relationships between iron overload, specific molecular pathways, and the detected signal alterations cannot be established in this cross-sectional study.

Despite the availability of ferritin and transferrin saturation data confirming systemic iron overload, NTBI concentrations and direct assessment of myocardial iron deposition, such as T2* cardiac MRI, were not available. Consequently, the relationship between cardiovascular signal alterations and myocardial iron burden could not be evaluated directly, and correlations with imaging-based measures of cardiac iron accumulation could not be performed.

In addition, the temporal relationships among autonomic dysfunction, cardiovascular variability, and potential arrhythmic or hemodynamic abnormalities could not be determined. Longitudinal studies incorporating repeated physiological assessments and direct measures of myocardial iron burden will be necessary to clarify the sequence of events and potential causal pathways.

Moreover, no direct measurements of respiratory function, gas exchange, tissue oxygenation, or oxygen transport were performed. Consequently, interpretations relating wavelet-derived oscillatory patterns to respiratory compensation, tissue perfusion, or oxygen-delivery-related mechanisms remain indirect and should be regarded as hypothesis-generating. Future studies incorporating respiratory monitoring, blood-gas analysis, or direct measures of tissue oxygenation will be required to verify these proposed mechanisms.

Medication use is a major limitation of the present study. A substantial proportion of HH patients were receiving antihypertensive and/or lipid-lowering medication, including angiotensin-converting enzyme inhibitors and statins, which may independently influence BP variability, autonomic regulation, vascular tone, and ECG-BP phase coherence. Excluding all medicated HH patients would leave only 16 HH participants, which was considered insufficient for robust frequency-specific group comparisons and would increase the risk of unstable estimates. Consequently, the present study cannot determine whether amplified BP oscillations or altered ECG-BP coherence are attributable to iron overload, medication effects, hypertension, and other comorbidities, or their interaction. The conclusions have therefore been restricted to exploratory associations observed in the full HH cohort.

## Figures and Tables

**Table 1:** Clinical characteristics, iron metabolism parameters, and comorbidities of the hereditary hemochromatosis cohort.

	HH All
HH cohort	30
Age, years	47 (25–71)
Male sex, n [%]	22 (73)
C282Y/C282Y genotype, n [%]	18 (60)
C282Y/H63D genotype, n [%]	7 (23)
H63D/H63D genotype, n [%]	3 (10)
C282Y/WT genotype, n [%]	2 (6)
Months from HH diagnosis	141 (36–288)
Laboratory parameters	
Iron [ug/dL]	183 (52–283)
Ferritin [ng/mL]	775 (13–4476)
Hemoglobin [mg/dL]	15.1 (13–18.2)
TSAT [%]	74 (42–100)
Glucose [mg%]	94 (75–123)
Aspartate aminotransferase (ASPAT) [U/L]	36 (10–105)
Alanine aminotransferase (ALAT) [U/L]	67 (21–238)
Concomitant diseases	
Hypertension, n [%]	12 (40)
Diabetes mellitus/impaired glucose tolerance (DM/IGGT), n [%]	13 (43)
Liver siderosis, n [%]	20 (66)
Liver cirrhosis, n [%]	16 (53)

## Data Availability

The data presented in this study are available on request from the corresponding author. The data are not publicly available due to privacy concerns.
